# Emergency Medicine Program Directors’ Perspectives on Changes to Step 1 Scoring: Does It Help or Hurt Applicants?

**DOI:** 10.5811/westjem.2021.3.50897

**Published:** 2021-12-20

**Authors:** Gabriella E. Glassman, Jennifer Black, Nicole Streiff McCoin, Brian C. Drolet

**Affiliations:** * Vanderbilt University Medical Center, Department of Plastic Surgery, Nashville, Tennessee; † Meharry Medical College, Pharmacy Department, Nashville, Tennessee; ‡ Ochsner Medical Center, Department of Emergency Medicine, New Orleans, Louisiana; § Vanderbilt University Medical Center, Department of Biomedical Informatics, Nashville, Tennessee

## Abstract

**Introduction:**

The United States Medical Licensing Examination (USMLE) Step 1 score is one of the few standardized metrics used to objectively review applicants for residency. In February 2020 the USMLE program announced that the numerical Step 1 scoring would be changed to a binary (Pass/Fail) system. In this study we sought to characterize how this change in score reporting will impact the application review process for emergency medicine (EM) program directors (PD).

**Methods:**

In March 2020 we electronically distributed a validated anonymous survey to EM PDs at 236 US EM residency programs accredited by the Accreditation Council for Graduate Medical Education.

**Results:**

Of 236 EM PDs, 121 responded (51.3% response rate). Overall, 72.7% believed binary Step 1 scoring would make the process of objectively comparing applicants more difficult. A minority (19.8%) believed it was a good idea, and 33.1% felt it would improve medical student well-being. The majority (88.4%) reported that they will increase their emphasis on Step 2 Clinical Knowledge (CK) for resident selection, and 85% plan to require Step 2 CK scores at application submission time.

**Conclusion:**

Our study suggests most EM PDs disapprove of the new Step 1 scoring. As more objective data is peeled away from the residency application, EM PDs will be left to rely more heavily on the few remaining measures, including Step 2 CK and standardized letters of evaluation. Further changes are needed to promote equity and improve the overall quality of the application process for students and PDs.

## INTRODUCTION

The United States Medical Licensing Examination (USMLE) Step 1 and Step 2 Clinical Knowledge (CK) have served as assessments of medical licensure eligibility.^1^ Over time, Step 1 has unintentionally guided undergraduate medical education curriculum and become a primary screening tool for objectively selecting applicants for residency interviews in many specialties.^2^–^4^ In an attempt to “reduce the adverse impact of the current overemphasis on USMLE performance in residency screening and selection,” the USMLE program announced in February 2020 that numerical Step 1 scoring would change to a binary system (Pass/Fail) no sooner than 2022.^1^

Every year US residency program directors (PD) are inundated with numerous applications for few positions.^5^–^7^ Emergency medicine (EM) is ranked as one of the top five specialties to which most US doctor of medicine (MD) and doctor of osteopathic medicine (DO) seniors match. In 2020, 9.5% and 11.4% of all matched US MD and DO seniors matched into EM, respectively.^6^ During the 2019–2020 application cycle, 3323 applicants applied for 2665 available EM postgraduate year-1 positions, and on average each EM PD reviewed 953 applications.^6^,^7^ Most programs receive far more applications than positions available, and program directors are forced to use metrics (eg, USMLE Step 1) to help filter and select applicants, even if those metrics are being used in an unintended manner.^2^

To address some of the shortcomings surrounding the review process, EM residency programs have deliberately implemented additional objective measures to standardize the review process. These measures include a standardized letter of evaluation (SLOE) and the previously piloted standardized video interviews (SVI). Overall, EM PDs have reported that Step 1 and Step 2 CK scores, SLOEs, and EM rotation grades are among the most critical determinants used to select applicants to interview.^5^–^8^ Altering Step 1 scoring could dramatically change the review process for residency programs. In fact, aggregate data from a recent national survey demonstrated resounding frustration from PDs in multiple specialties.^9^ This study applies additional scrutiny to the perspectives of EM PDs who, on average, review approximately 1000 applications per cycle.^7^

## METHODS

After institutional review board exemption was granted, we invited PDs from Accreditation Council for Graduate Medical Education (ACGME)-accredited residency programs to participate in an anonymous, validated survey using Research Electronic Data Capture (REDCap, Vanderbilt University, Nashville, TN). The survey instrument underwent pre-pilot testing and was piloted with a group of 27 academic physicians. We assessed internal validity by computation of Cronbach’s alpha (0.87). No modifications were made after pilot testing was performed. The 19-item survey was electronically distributed to all PDs of ACGME-accredited residency programs in 30 specialties, including EM. In disseminating the survey to EM PDS we used the email addresses of 236 EM PDs (92.2% of all EM PDs), which we obtained from a publicly available ACGME listing of accredited programs during the academic year 2019–2020. Each unique email represented an EM PD from a separate EM residency program. We sent three subsequent survey requests to non-responders before the analysis was completed in an effort to generate greater participation.

The anonymous REDCap survey consisted of an optional demographic collection segment, a required series of three-point Likert scale questions (ie, disagree, neutral, agree), and an optional free-response comment box at the conclusion of the section. Survey items were designed in such a way that disallowed submission if required data collection fields were absent or incomplete. For this study’s purpose, the response rate was determined by the overall number of submitted surveys received (partial or completed) compared to the initial number of survey requests sent. We calculated descriptive statistics using Microsoft Excel (Microsoft Corporation, Redmond, WA).

**Population Health Research Capsule**

What do we already know about this issue?

*In 2020, the United States Medicine Licensing Examination changed its Step 1 from a numerical to binary scoring system. Most residency program directors (PD) disapprove of this change*.

What was the research question?

*Do Emergency Medicine residency PDs approve of the change to Step 1 scoring?*

What was the major finding of the study?

*Most Emergency Medicine PDs disapprove of the pass/ fail Step 1 scoring system. As a result, more PDs will increase their emphasis of Step 2 Clinical Knowledge scores*.

How does this improve population health?

*More standardized metrics are needed in the residency application process to increase equity*.

## RESULTS

In March 2020, 121 of 236 EM PDs responded to a REDCap survey (51.3% response rate). The majority (67%) of respondents were male, with a mean tenure as PD of 5.8 ± 5.4 years (n = 105). Over half (61.9%) of the responding PDs had one to five years of experience while 37.1% had greater than five years of experience. In total 13.3% had held their positions for 10 or more years. Only one response (0.01%) came from a PD with less than one year of experience. Among those who responded, 26.7% were from programs in the Northeast (47.8% of respondents from that region); 35.8% in the South including Puerto Rico (58.1% of respondents from that region); 24.2% in the Midwest (45.3% of respondents from that region); and 13.3% in the West (51.6% of respondents from that region).

**Figure 1:**
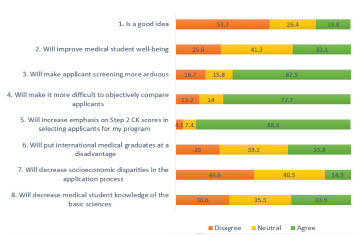
Program directors’ responses to the planned use of binary scoring for USMLE* Step 1. *United States Medical Licensing Exam. *CK*, clinical knowledge.

Of all the survey responses received, 19.8 % of PDs agreed that the scoring change was a good idea and 33.1% believed it would improve medical student well-being. Additionally, 67.5% anticipated the change would make applicant screening “more arduous,” and 72.7% felt it would be more difficult to compare applicants objectively. Most PDs (88.4%) reported that binary Step 1 scoring would increase their emphasis on USMLE Step 2 CK scores. Furthermore, 35.8% believe this change will disadvantage international medical graduates applying to EM. Only 14.9% felt this change would decrease socioeconomic disparities among applicants (Figure 1).

**Figure 2:**
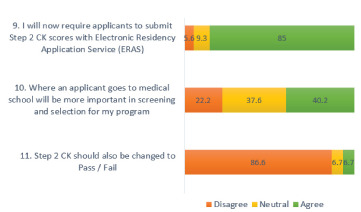
How program directors plan to adjust their applicant screening process. *CK*, clinical knowledge.

As a result of changing USMLE Step 1 to Pass/Fail, the majority (85%) of EM PDs indicated that they plan to require Step 2 CK scores to be submitted at the time of application. Additionally, 40.2% of the PDs reported that medical school reputation would become more critical for the selection process. Only 6.7% of PDs recommended changing Step 2 CK to Pass/Fail (Figure 2).

Of the 121 surveys we received 33 had free-text responses. Two authors (GEG and JB) reviewed and subjectively ranked these responses based on positivity, neutrality, or negativity. Of these responses, four expressed a favorable opinion of the change (12.1%), seven remained neutral (21.2%), and 22 were negative (66.7%).

The positive comments from PDs in favor of the change mostly focused on the long misuse of Step 1 scores to filter students and the potential for bias against students from under-represented groups. All four comments indicated that the use of Step 1 scores to compare students was not an ideal method and favored a preference for continued reforms to student evaluation. As stated by one PD, “A single exam score does not accurately depict the student’s qualifications as a whole. Too much emphasis is placed on this score. We ought to utilize additional measures on equal footing as the muscle score. For example, a letter of recommendation, Dean’s letter, transcript, interview, etc.” Another PD wrote, “… I also think it was discriminatory toward certain socioeconomic groups. The same may be true for [S]tep 2 though I think it predicts more for emergency medicine.”

Neutral remarks often focused on other potential screening methods. As one PD reported, “While we had Step 1 scores listed as part of the criteria, our other factors have always carried much more weight.” Other comments shared a desire for a different ranking system other than Pass/Fail scoring, such as “strict class ranking systems or transitioning Step 1 to measuring quartiles or thirds.”

Given the higher percentage of negative responses, there was a more significant variation in responses. In one PD’s words, “this is a bad idea and hampers residency programs’ ability to objectively compare applicants from different medical schools.” Most commentaries indicated the PD’s plan to transition to using Step 2 as a new marker for granting student interviews and away rotations. Eight commenters described the change as a “bad idea,” with two calling it “ridiculous’ or “not logical.” Two even requested to reverse the change. Another PD wrote, “I see overinflated grades at medical schools, which makes a standardized test important. This is a step back. Students must learn medicine as they are taking care of people’s lives. A few exams require preparation and acquisition of knowledge; it doesn’t lead to burnout. The thought process behind it is understood, but the conclusion and plan are wrong.”

## DISCUSSION

In this study we found that most EM PDs disagree with the newly established binary Step 1 scoring system. The rationale supporting the new format released in the USMLE Summary Report defined five specific areas that would be enhanced by the change. According to these guiding principles, the adoption of a Pass-Fail system is intended to “address flaws in the transition from undergraduate to graduate medical education systems, improve reliability of assessments in medical education, promote holistic review of residency applicants, maintain quality and integrity in the US medical licensure system for both domestic and international graduates, and ultimately, improve examinee and physician well-being.”^7^ Unfortunately, among those EM PDs who responded to our survey, only 33.1% of PDs believed that medical student well-being would improve. Furthermore, 88.4% of respondents indicated they were planning to increase emphasis on USMLE Step 2 CK scores as a countermeasure, compared to the 48% of programs requiring USMLE Step 2 CK scores in a 2018 report.^5^ This represents a significant shift in focus and suggests that the current emphasis on standardized testing will merely be moved from Step 1 to Step 2 CK, rather than be mitigated as was initially intended.

While this survey reflects the current opinions of EM PDs and the actual determinants for interview invitations may vary, the impact on future applicants cannot be ignored. Many US MD and DO seniors currently delay taking the Step 2 CK exam to prioritize away or audition rotations. This shift in focus from Step 1 to Step 2 CK could dramatically change a medical student’s curriculum, particularly during their third and fourth years.

Numeric Step 1 scores have anecdotally been used to compare Step 2 CK performance and gauge a student’s improvement over time. Without a numerical Step 1 score, Step 2 CK scores are reduced to a single data point, rather than a trend. In 2018, Negaard et al found only 10% of EM residency educators required a USMLE Step 1 score greater than 220, and that most required a minimum score of 200–210 or a passing grade.^11^ Despite the small percentage of programs that required a target score of 220 for screening, now 72.7% of PDs believe the absence of the Step 1 score will make it more difficult to compare students’ academic achievements objectively. This may imply that even though a target score was not required, the objective metric provided by a standardized test was still a valuable component of the evaluation process.

In the last 25 years, EM residencies have recognized the need for objective data in the applicant review process. The Council of Emergency Medicine Residency Directors implemented a template for standardized letters of recommendation in 1997, now referred to as the SLOE. This metric has been shown to increase efficiency, eliminate the potential for inflated student evaluations, and to have a higher degree of inter-rater reliability than traditional narrative letters of recommendation.^12^ Before the COVID-19 pandemic and subsequent travel restrictions, 80% of EM programs required at least one SLOE to be considered for an interview.^11^ To acquire a SLOE, a medical student must rotate at a designated SLOE-approved institution. Restrictions on the number of away rotations performed during the COVID-19 pandemic meant there were subsequently fewer metrics for comparison included in the applications.

In addition to the SLOE, the Association of American Medical Colleges launched a pilot for the SVI in 2017 as another tool to provide additional standardized data to EM PDs. Although this pilot has concluded, it does highlight the need for more standardized data to aid EM PDs in the review of residency applications. The removal of numerical Step 1 scores would mean that EM PDs have even less objective data to base their decisions for interview invitations. This unfortunately comes in an era where the push to create more objective measures, such as the SLOE and SVI, has clearly been sought and created iwithin the field of EM.

## LIMITATIONS

This survey was distributed to PDs only and may not reflect all the EM graduate medical education community. Further, while email addresses were collected from publicly available documents, not all PDs’ emails were available. Therefore, we were unable to query all active EM PDs. Our study’s response rate was 27.5% greater than the ACGME’s PD survey of the 2019–2020 application cycle.^7^ We cannot reasonably perform a non-response bias analysis; however, it remains possible that PDs interested in the topic were more inclined to respond. More responses were obtained from male PDs; this was expected as the EM field is predominately male with women comprising only 27.6% of active US emergency physicians in 2017.^13^ Moreover, in 2011 Long et al found that 18.8% of PD positions were held by female phsyicians.^14^ This indicates that our pool of mostly male respondents is roughly similar to the overall population of EM PDs in relation to gender. The free-response comments section was completed by only 27% (33/121) of the survey respondents. Lastly, this survey did not investigate the PDs’ backgrounds or their level of involvement in undergraduate medical education. This may present a potential confounder.

## CONCLUSION

We found that most EM program directors do not favor the move to binary USMLE Step 1 scoring. Our study suggests that the proposed change in USMLE score reporting may not achieve its intended goal of reducing overall emphasis on USMLE performance. Program directors may merely shift their focus from one standardized exam to another. The present study suggests that changes to Step1 scoring may not decrease disparities, and further research will be needed to assess the true effects.
